# Porcine epidemic diarrhea virus S1 protein is the critical inducer of apoptosis

**DOI:** 10.1186/s12985-018-1078-4

**Published:** 2018-11-07

**Authors:** Yifeng Chen, Zhibang Zhang, Jie Li, Yueyi Gao, Lei Zhou, Xinna Ge, Jun Han, Xin Guo, Hanchun Yang

**Affiliations:** 1 Key Laboratory of Animal Epidemiology of the Ministry of Agriculture, College of Veterinary Medicine and State Key Laboratory of Agrobiotechnology, China Agricultural University, No.2 Yuanmingyuan West Road, Haidian Distract, Beijing, 100193 People’s Republic of China; 2 Animal Medicine Research Center of DBN Group, South Crossroad of Xiangrui Street and Huatuo Road DBN Daxing Science Park, Daxing Distract, Beijing, 102600 People’s Republic of China

**Keywords:** Porcine epidemic diarrhea virus (PEDV), Spike S1 protein, Apoptosis, Apoptosis-inducing factor mitochondria associated 1 (AIFM1)

## Abstract

**Background:**

Porcine Epidemic Diarrhea (PED) is an acute and highly contagious enteric disease caused by PED virus (PEDV), characterized by vomitting, watery diarrhea and fatal dehydration with high mortality in sucking piglets of one week of age. Although PEDV induced cell apoptosis has been established in vitro and in vivo, the functional protein that contributes to this event remains unclear.

**Methods:**

The activation or cleavage of main apoptosis-associated molecular such as AIFM1, caspase-3, caspase-8, caspase-9 and PARP in PEDV infected host cells were analyzed by western blotting. The nuclear change of infected cell was monitored by confocal immunofluorescence assay. The overexpressing plasmids of 16 non-structural proteins (Nsp1–16) and 6 structural proteins (M, N, E, ORF3, S1 and S2) were constructed by cloning. Cell apoptosis induced by PEDV or overexpression non-structural or structural proteins was measured by the flow cytometry assay.

**Results:**

PEDV could infect various host cells including Vero, Vero-E6 and Marc-145 and cause obvious cytopathic effects, including roundup, cell fusion, cell membrane vacuolation, syncytium formation and cause apparent apoptosis. In infected cells, PEDV-induced apoptosis is accompanied by nuclear concentration and fragmentation as a result of caspase-3 and caspase-8 activation and AIFM1 and PARP cleavage. Overexpression of S1 Spike protein of PEDV SM98 strain effectively induced host cell apoptosis, while the expression of the other non-structure proteins (Nsp1–16) and structural proteins (M, N, E, S2 and ORF3) has no or less effect on cell apoptosis. Similarly, expression of S1 protein from wild-type strain BJ2011 or cell-adapted strain CV777, also induce apoptosis in transfected cells. Finally, we demonstrated that the S1 proteins from various coronavirus family members such as TGEV, IBV, CCoV, SARS and MERS could also induce Vero-E6 cells apoptosis.

**Conclusion:**

S1 Spike protein is one of the most critical functional proteins that contribute to cell apoptosis. Expression of S1 proteins of the coronavirus tested in this study could all induce cell apoptosis suggesting S1 maybe is an effective inducer in Coronavirus-induced cell apoptosis and targeting S1 protein expression probably is a promising strategy to inhibit coronavirus infection and thus mediated apoptosis on host cells.

**Electronic supplementary material:**

The online version of this article (10.1186/s12985-018-1078-4) contains supplementary material, which is available to authorized users.

## Background

Porcine Epidemic Diarrhea (PED) is an acute and highly contagious enteric disease characterized by severe watery diarrhea, dehydration, and anorexia. Deceased piglets presented with thin and almost transparent small intestines containing undigested milk curdles. The etiological agent PED virus (PEDV) was first isolated and recognized from Europe in the 1970s [1, 2], then it was spread and prevalent in Asian for decades [3, 4]. PEDV originally caused a relatively mild and sporadic disease. However, since more virulent variant strains appeared in 2010 [5–8], PEDV has been subsequently associated with severe outbreaks of diarrheal disease [9] in Asia and in North American [10–13]. Acute PEDV outbreaks normally resulted in enormous economic losses to swine industries around the world, for instance, in 2013 to 2014 PEDV killed more than 7 million pigs in the North American [14]. Currently, PEDV poses a serious threat to the swine industry worldwide.

PEDV is an enveloped single-stranded and positive-sense RNA virus, belongs to the genus Alpha coronavirus, family Coronaviridae, order Nidovirales [2]. The genome of PEDV is about 28 kb and comprises of a 5′ untranslated region (UTR), at least 7 open reading frames (ORF1a, ORF1b, and ORF2–6), and a 3’ UTR. The ORF 1a and 1b cover the 5′-proximal two-thirds of the genome coding for replicase polyprotein (pp) la and pp1ab, respectively [15, 16]. These pp1a and pp1ab can be cleaved by internal proteases generating 16 nonstructural proteins, namely nsp1–16. ORF2–6 encode four structural proteins including the spike (S), envelope (E), membrane (M) and nucleocapsid (N) proteins, while ORF3 encodes an accessory protein [15]. The large spikes on the coronavirus envelope are composed of trimers of the spike proteins. The spike protein mediates viral entry into host cells by functioning as a class I viral fusion protein [17]. During maturation, the spike protein is often cleaved into a receptor-binding subunit S1 and a membrane-fusion subunit S2 [18].

PEDV induce infected host cell apoptosis has been established in vitro and in vivo [19]. PEDV also induces caspase-independent apoptosis in host cells by the activation of mitochondrial apoptosis-inducing factor based on the observation of AIFM1 relocated into the nucleus during the PEDV infection [19]. However, the protein that is mainly responsible for this induction remains unclear. In this study, we have provided evidence that S1 protein is the main inducer for cell apoptosis during PEDV infection.

## Methods

### Cells and viruses

Vero, Vero-E6, MARC-145 and BHK-21 cells were obtained from American Type Culture Collection (ATCC), all the cells were maintained in Dulbecco Modified Eagle Medium (DMEM; Invitrogen) supplemented with 10% fetal bovine serum (FBS) and antibiotics (100 U/ml of penicillin and 100 μg/ml of streptomycin) in a 5% CO_2_ incubator. PEDV SM98 and CV777 strains were kept in our laboratory, wild-type strains BJ2011 was isolated from a farm in Beijing in 2011.

### Reagents

Fluorescein (FITC)-conjugated affiniPure Goat anti-Mouse IgG (H + L) was a product of Jackson Immuno Research; Anti-PEDV Spike mAb, Annexin V-PE/7-AAD Apoptosis Detection Kit was purchased from BD; Caspase-3, caspase-8, caspase-9 and PARP rabbit mAbs were bought from Cell Signaling Technology; Anti-AIFM1 mice mAb was a product of Sigma-Aldrich; Caspase-3, − 8 and − 9 activity assay kits were purchased from Biovision.

### Plasmids construction

The Nsp1–16, M, N, E, ORF3, S1 and S2 genes were cloned from PEDV strain SM98 using specific primers (Additional file 1: Table S1). Primers were synthesized by BGI Tech Solutions Co., Ltd. (Beijing, China). All the plasmids were constructed by standard recombined DNA techniques. Briefly, all the non-structural and structural genes were amplified by RT-PCR from PEDV RNA genome and cloned into the pEGFP-N1 vector. The S1 genes of transmissible gastroenteritis virus (TGEV), avian infectious bronchitis virus (IBV) and canine coronavirus virus (CCoV) were cloned from vaccine strains (TGEV H Strain, IBV-H52 strain and CCoV K378 strain, respectively). The S1 genes of Middle East respiratory syndrome (MERS) coronavirus and Severe acute respiratory syndrome (SARS) coronavirus were synthesized by Genscript according to the sequences submitted in Genebank (accession number: KY581693.1 and AH013709.2, respectively).

### Apoptosis assay

Vero, Vero-E6 and Marc-145 cells were seeded in six-well-plate and cultured overnight, cells were then mock-infected or infected with PEDV CV777 or SM98 strains at a multiplicity of infection (MOI) of 0.1 for 48 h. Cells were trypsinized and resuspended with buffer (135 mM NaCl, 10 mM HEPES, 5 mM CaCl_2_) and stained with Annexin V-PE and 7-AAD at room temperature for 15 min. Cells were analyzed by flow cytometry. Fluorescence-activated cells sorter (FACS) data were analyzed using CellQuest software (BD).

Vero-E6 or BHK21 cells were cultured in six-well-plate and grow to 70–80% confluent, cells were transfected with various plasmids including pEGFP-N1, pEGFP-Nsp1–16, pEGFP-M, pEGFP-N, pEGFP-E, pEGFP-ORF3, pEGFP-S1 and pEGFP-S2 by Lipofectamine LTX. After 48 h transfection, cells were harvested and stained with Annexin V-PE and 7-AAD at room temperature for 15 min. GFP-positive cells were gated for apoptosis analysis. FACS data were analyzed with CellQuest software (BD).

### Western blotting

Protein samples were separated by SDS-PAGE and were electrically transferred onto a polyvinylidene fluoride (PVDF) membrane. After being blocked with 5% skim milk in phosphate-buffered saline (PBS), the membrane was incubated with proper antibodies and subsequently probed with appropriated horseradish peroxidase (HRP) conjugated goat anti-mouse secondary antibody. The protein bands were developed with the ECL western blotting system (Fisher Scientific) and exposed to a fluorchem E apparatus (Proteinsimple, Santa Clara, CA, USA). Western blotting bands are quantified according to intensity by using ImageJ software, normalized to β-actin and expressed relative to mock infection.

### Confocal microscopy assays

Vero cells were grown on coverslips in 24-well-plate (Costar, Corning Incorporation) and infected with the SM98 virus at an MOI of 0.1, uninfected cells serve as a mock control. The cells were fixed and permeabilized with cold anhydrous ethanol for 20 min at room temperature (RT) at 24, 48 and 72 h post infection, respectively. Followed by being blocked with 2% BSA in PBS for 1 h at RT. Then, the cells were incubated with mice anti-PEDV S monoclonal antibody at 4 °C in a humid chamber. After being rinsed 3 times with PBS, the cells were incubated with fluorescein isothiocyanate (FITC)-conjugated goat anti-mice antibody for 1 h at RT. Finally, the cells were stained with DAPI, and the images were viewed under an Olympus confocal microscope (fluoview 1000×).

### Caspase-3, − 8 and − 9 activity assays

Vero cells were seeded on six-well-plate and were mock-infected or infected with SM98 strain at an MOI of 1. Infected cells were incubated for 48 h before trypsinized. Cells were washed with cold PBS, resuspended in 50 μL of chilled cell lysis buffer, and incubated on ice for 15 min. The cell lysates were centrifuged at 10,000×g for 15 min. The supernatants were collected and frozen at − 70 °C, the activities of caspase-3, − 8 and − 9 in samples were measured by colorimetric assay with a plate reader.

### Statistical analysis

The statistically significant differences between PEDV infected cells and controls in the induction of apoptosis and caspase activation and, between the recombinants plasmid transfected cells and pEGFP-N1 transfected control in the induction of apoptosis was determined by unpaired T-tests or ANOVA accordingly with GraphPad Prism (Version 5.0) software. Differences were considered statistically at a value of *p* < 0.05.

## Results

### Cytopathogenic effect produced by PEDV strains SM98 and CV777 infection

**Fig. 1:**
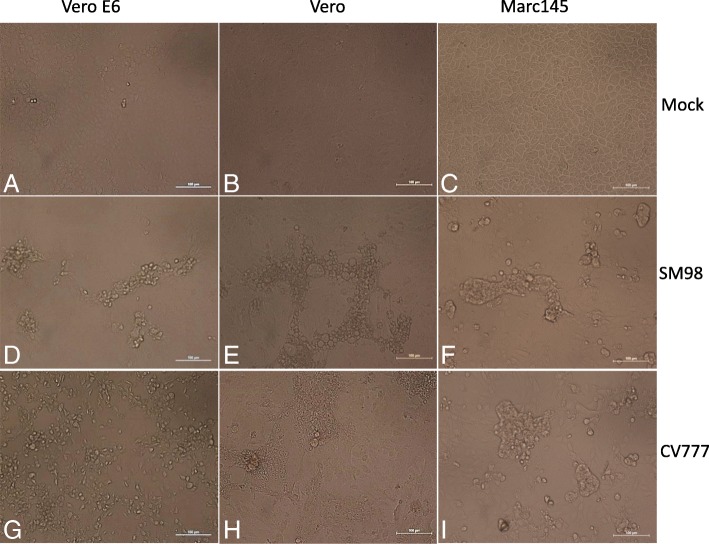
PEDV infection results in obvious cytopathic effects.(**a, b, c**) Vero-E6, Vero and Marc-145 cells mock control. (**d**, **e**, **f**) CPE of Vero-E6, Vero and Marc-145 cells infected with SM98. (**g**, **h**, **i**) CPE of Vero-E6, Vero and Mark-145 cells infected with CV777

When PEDV SM98 and CV777 strains were inoculated into Vero, vero-E6 and Mark-145 cells respectively, distinct cytopathic effects (CPEs) including roundup (Fig. 1 d and g), cell fusion (F and I), vacuolation (E), syncytium (H) and detachment were observed, as shown in Fig. 1.

### PEDV infection induces apoptosis in multiple host cells

**Fig. 2:**
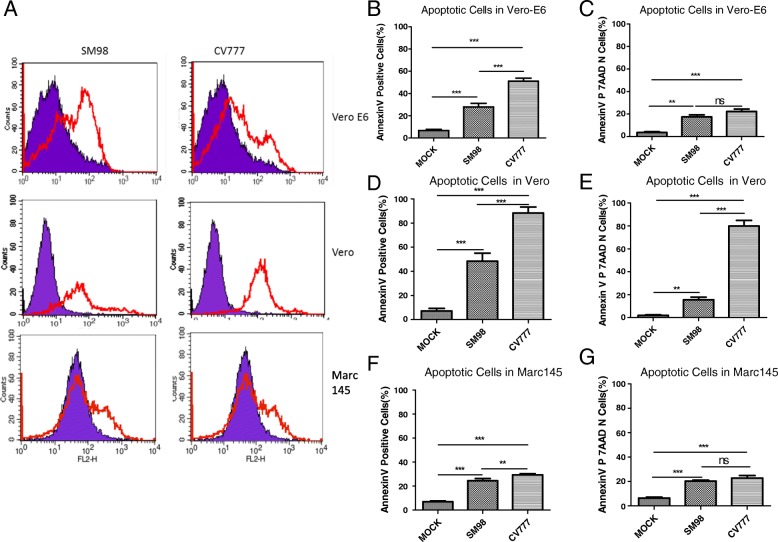
PEDV infection results in Apparent Apoptosis in Vero, Vero-E6 and MARC145 cells. Mock and infected Vero, Vero-E6 and MARC145 cells were collected and stained with 7-AAD and annexin-V-PE at 48 h post infection, and then analyzed by flow cytometry. (**a**) PEDV SM98 and CV777 strains both could induce significant apoptosis in Vero-E6, Vero and MARC-145 cells. (**b, d, f**) Percentages of annexin-V-PE positive cells from SM98 and CV777 infected Vero-E6, Vero and Marc-145 cells. (**c**, **e**, **g**) Percentages of annexin-V-PE positive and 7AAD negative cells from SM98 and CV777 infected Vero-E6, Vero and Marc-145 cells. Results are representative of three independent experiments. Data are represented as mean ± SD, *n* = 3. (**, *p* < 0.01; ***, *p* < 0.001;ns, no significant).

Mock and infected Cells were collected at 48 h post-infection and stained by Annexin V-PE/7-AAD and analyzed by flow cytometry. As shown in Fig. 2a, SM98 and CV777 strains infection induce significant apoptosis in Vero, Vero-E6 and Marc-145 cells, no matter total apoptosis or early apoptosis. Percentages of Annexin-V-PE positive cells from SM98 and CV777 infected Vero-E6, Vero and Marc-145 cells are shown in Fig. 2b, d and f respectively, while percentages of Annexin-V-PE positive and 7AAD negative cells are shown in Fig. 2c, e and g.

### PEDV infection resulted in syncytium formation and cell nuclear DAN fragmentation and chromatin condensation

**Fig. 3:**
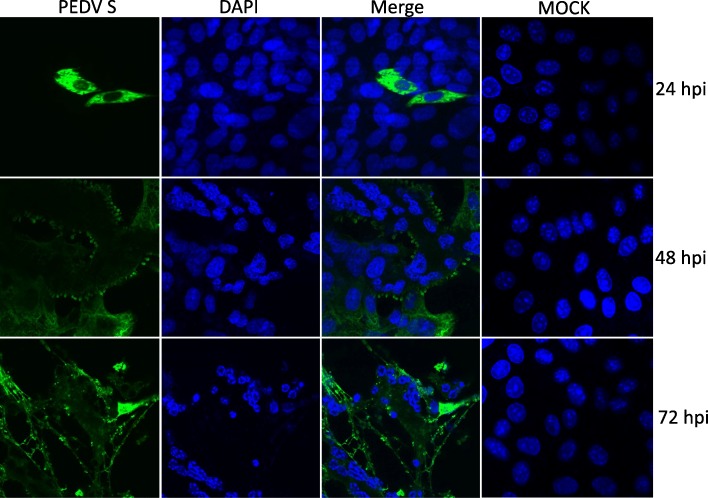
PEDV infection results in cell nuclear DAN fragmentation and chromatin condensation. Vero cells grown on coverslips were infected with PEDV SM98 at a multiplicity of infection of 0.1, The cells were fixed and incubated with mice anti PEDV S monoclonal antibody and DAPI at 12, 48 and 72 h post infection. (fluoview 1000×)

The nuclear change of PEDV infected cells was monitored by confocal immunofluorescence assay. As can be seen in Fig. 3, with the PEDV replication, the nuclear of infected cells gradually gathered, piled up, some of which split into fragments. These gathered nuclears and nuclear fragments and the surrounded cytoplasmic membrane formed a multinucleated syncytium. At the end stage of infection (72 h post-infection), the nuclear of infected cell split into condensed fragments completely.

### PEDV infection activates AIFM1, Caspase-8 and Caspase-3

**Fig. 4:**
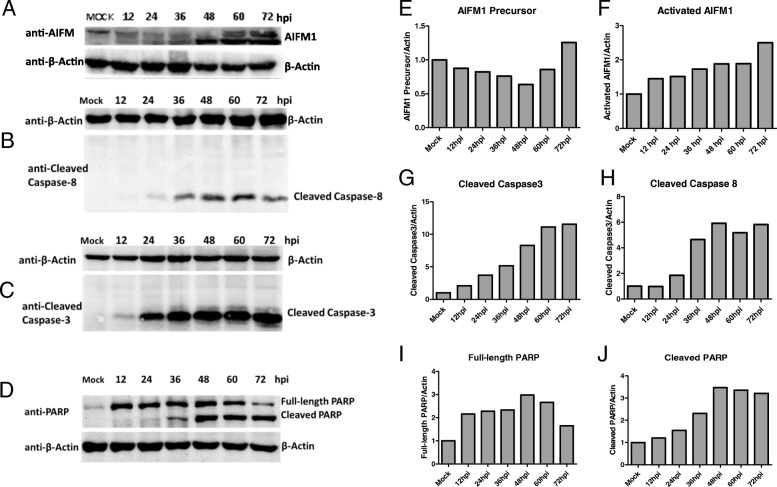
PEDV infection activates AIFM1, Caspase-3 and Caspase-8. Mock and infected Vero cells were collected at 12, 24, 36, 48, 60 and 72 h post infection and split with RIPA lysis buffer and supersonic. (**a-d**) Cleaved AIFM1, Caspase-3, Caspase-8 and PARP were analyzed by western blotting. (**e-j**) The graphs show the quantification of WB bands in panel A-D by testing optical density using ImageJ - system, normalized to β-actin and expressed relative to mock infection. Two biological replicated experiments were performed for each protein, and one result was represented

To investigate the molecular mechanism of PEDV-induced apoptosis in host cells, we detected the time-course activation status of AIFM1, caspase-3, caspase-8 in PEDV-infected Vero cells. The results showed that AIFM1 was cleaved upon PEDV infection. The amount of truncated AIFM1 was increased gradually (Fig. 4a). The cleaved caspase-8 was also detected at 24 h post infection (B), while the cleavage of caspase-9 was not observed in the whole course. Consistent with AIFM1 and caspase-8 activation, the downstream effector caspase-3 (C) and substrate PARP (D) were also cleaved in response to PEDV infection. This together suggested PEDV infection not only activates AIFM1 but also induces the extrinsic apoptotic pathway activation. The relative changes of Western blot band intensity of AIFM1 precursor and activated AIFM1, as well as the cleaved caspases-3, − 8 and PARP to β-actin were shown in Fig. 4e -j.

**Fig. 5:**
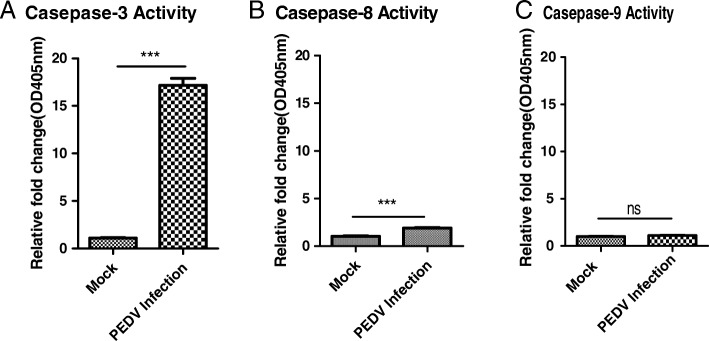
PEDV infection results in the activation of Caspase-3 and Caspase-8,but not Caspase-9. The activation of Caspase-3, Caspase-8 and Caspase-9 were detected by the Caspase activity assay kits. The relative fold change (OD405nm) of Caspase-3, − 8 and − 9 activities are shown with graphs. Data are represented as mean + /− SD, *n* = 3.(***, *p* < 0.001;ns,no significant)

The effect of PEDV infection on caspase-3, − 8 and − 9 activations was further confirmed by using caspase activity assay kits at 48 h post infection. The relative fold changes (OD_405_nm) of caspase-3, caspase-8 and caspase-9 in the PEDV infected cell lysis were shown in Fig. 5. The results showed that PEDV infection results in obviously Caspase-3 activation and caspase-8 activation, but no activation of caspase-9. The results are also consistent with above western-blot results.

### SM98 strain expression plasmids construction and fusion protein fluorescent identification

To investigate the functional gene that is responsible for PEDV-induced apoptosis. We cloned all 16 non-structural genes (Nsp1–16) and 6 structural genes including M, N, E, ORF3, S1 and S2 from PEDV SM98 strain and inserted them into the pEGFP-N1 vector. These recombinant plasmids were identified by sequencing and fluorescence microscopy. The bright green fluorescence was observed from these recombinant plasmids-transfected cells, although the expression efficiency of each recombinant plasmid is different due to individual gene specificity (Additional file 2: Figure S1 and Additional file 3: Figure S2). This indicated that these PEDV structural and non-structural genes are correctly constructed and fused well with the EGFP gene.

### S1 protein is a critical inducer of apoptosis in host cells

**Fig. 6:**
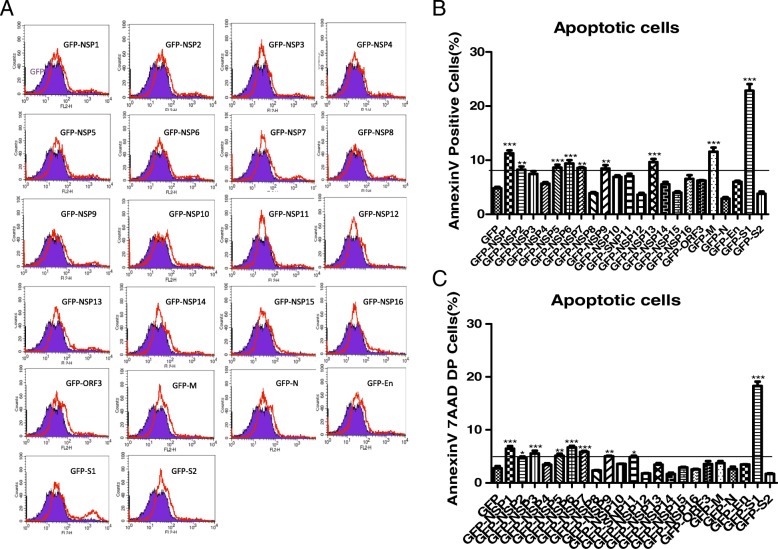
PEDV S1 Protein is the Critical inducer of apoptosis among all the Structural and Nonstructural Proteins. pEGFP-N1 and all the recombinant plasmids were transfected into confluent Vero E6 monolayer cells respectively. 48 h later, the cells were harvested and analyzed by Fluorescence-activated cells sorter (FACS), GFP-positive cells were gated for apoptosis analysis. (**a**)The results showed that PEDV S1 Protein is the Critical inducer of Apoptosis in host Cells. (**b**) Percentages of annexin-V-PE positive cells from gated cells. (**c**) Percentages of annexin-V-PE and 7AAD double positive cells from gated cells. Results are representative of three independent experiments. Results are representative of three independent experiments. Data are represented as mean + /− SD, n = 3. (*, *p* < 0.05; **, *p* < 0.01; ***, *p* < 0.001)

Next, we detected the effect of the individual gene on cell apoptosis. The empty pEGFP-N1 vector and the recombinant plasmids infusion with the nonstructural proteins (Nsp1–16) and structural proteins (M, N, E, S1, S2 and ORF3) were transfected into 90% confluent Vero E6 monolayer cells separately. 48 h after transfection, cells were stained with AnnexinV-PE and 7-AAD and detected by fluorescence-activated cells sorter (FACS), GFP-positive cells were gated for apoptosis analysis. Among all the structural proteins of PEDV SM98 strain, S1 protein has the predominant capability to induce apoptosis while nuclear protein N, spike protein S2 and ORF3 have no effect on apoptosis induction. Membrane protein (M) and Nonstructural protein Nsp1, Nsp3, Nsp5, Nsp6 and Nsp7 also induce cell apoptosis but to variable less extents, as shown in Fig. 6a. Percentages of Annexin-V-PE positive cells from gated cells are shown with graphs in Fig. 6b. Percentages of Annexin-V-PE and 7AAD double positive cells from gated cells are shown with graphs in Fig. 6c. This suggests that S1 protein probably is the most critical protein-mediated PEDV-induced apoptosis.

### The S1 proteins in new-emerging and cell adapted PEDV strains also induce cell apoptosis

**Fig. 7:**
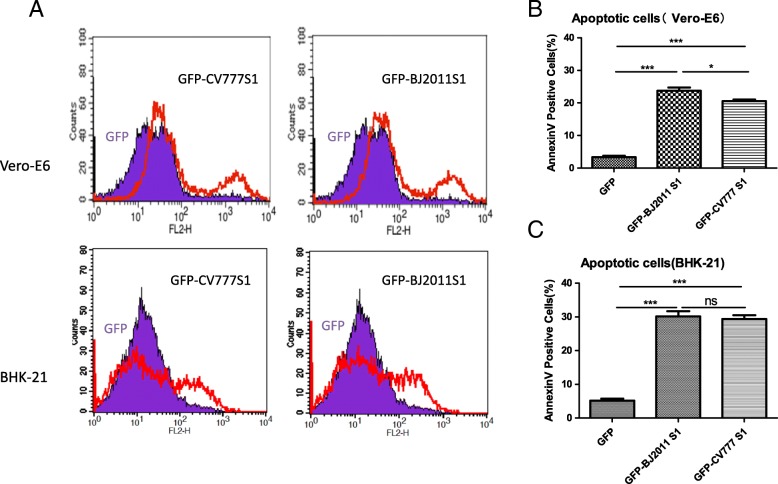
PEDV CV777 and new-emerging strain BJ2011 S1 protein Could also induce apoptosis in VERO-E6 even in BHK-21 cells. pEGFP-N1 and the recombinant plasmids expressing CV777 or BJ2011 S1 protein were transfected into confluent Vero-E6 or BHK-21 monolayer cells respectively. 48 h later, the cells were harvested and analyzed by Fluorescence-activated cells sorter (FACS), GFP-positive cells were gated for apoptosis analysis. (**a**) PEDV CV777 and new-emerging strain BJ2011 S1 protein Could also induce apoptosis in Vero-E6 even in BHK-21 cells. (**b**) Percentages of annexin-V-PE positive Vero-E6 Cells from gated cells are shown with graphs. Results are representative of three independent experiments. (**c**) Percentages of annexin-V-PE positive BHK-21 Cells from gated cells are shown with graphs. Results are representative of three independent experiments. (*, *p* < 0.05; **, *p* < 0.01; ***, *p* < 0.001; ns, no significant)

Next, we asked whether the S1 protein of other different PEDV strains could also induce apoptosis in different host cells. The S1 genes of CV777, a cell-adapted PEDV strain, and BJ2011, a new emerging wild-type PEDV isolated from Beijing in 2011 (The relationship of these PEDV S proteins has been analyzed and reported previously in our laboratory) [20], were constructed in fusion with EGFP in pEGFP-N1 vector. Surprisingly, transfection S1 gene of CV777 and BJ2011 effectively induce monolayer Vero-E6 cells apoptosis. The similar result was also observed in transfected BHK-21 cells (Fig. 7a). Collectively, this result suggested that S1 protein is the determinant protein in PEDV infection-induced cell apoptosis, and this function is less dependent on virus biological or genetic differences. Percentages of apoptotic cells are shown with graphs in Fig. 7b and c respectively. This suggested that S1 protein probably is the most critical protein-mediated PEDV-induced apoptosis.

### The S1 protein from various coronavirus resources induce apoptosis in Vero-E6 cells

**Fig. 8:**
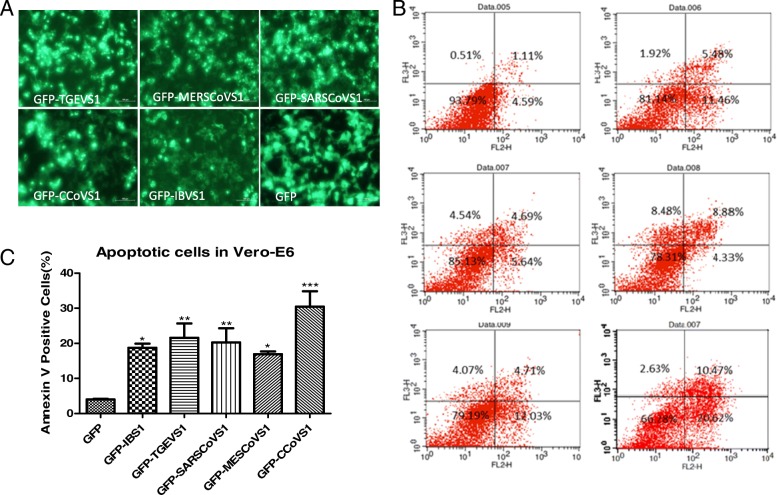
TGEV, IBV, CoCoV, SARS and MERS CoV Spike Protein S1 Could also Induce Apoptosis in Vero-E6 cells. TGEV, IBV, CCoV, SARS and MERS S1 genes were cloned or synthesized in fusion with EGFP gene and were transfected into VERO-E6, 48 h later, the cells were harvested and analyzed by Fluorescence-activated cells sorter (FACS), GFP-positive cells were gated for apoptosis analysis. (**a**) TGEV, IBV, CCoV, SARS and MERS S1 genes were correctly constructed in fusion with EGFP. The empty vector pEGFP-N1 and the recombinant plasmids were all transfected into confluent BHK-21 monolayer cells separately. 24 h later, bright green fluorescence was observed from the all the recombinant plasmids transfected cells. (**b**) The results showed that TGEV, IBV, CCoV, SARS and MERS S1 Protein Could Induce Apoptosis in Vero-E6 cells. (**c**)Percentages of annexin-V-PE positive cells from gated cells are shown with graphs. Results are representative of three independent experiments. (*, *p* < 0.05; **, *p* < 0.01; ***, *p* < 0.001)

Finally, we explored the pro-apoptotic function of the S1 protein in other coronavirus such as transmissible gastroenteritis virus (TGEV), canine coronavirus (CCoV), avian infectious bronchitis virus (IBV), severe acute respiratory syndrome coronavirus (SARS-CoV) and middle east respiratory syndrome coronavirus (MERS-CoV). The S1 gene from these coronaviruses was successfully cloned or synthesized, and infused with EGFP in pEGFP-N1 vector (Fig. 8a). The apoptosis potential of this S1 protein was analyzed by FACS. As shown in Fig. 8b, S1 protein from all these coronaviruses could induce Vero-E6 cells apoptosis (Fig. 8c). Altogether, these data suggested that S1 protein have a general function inducing cell apoptosis in the coronaviruses, targeting S1 protein probably is a promising strategy to inhibit coronavirus infection and thus decreases the threat to animal industry and human health.

## Discussion

In this study, we observed that PEDV SM98 and CV777 strains induce apoptosis in Vero, Vero-E6 and Marc-145 cells with distinct cytopathic effects (CPEs) including roundup, cell fusion, vacuolation, syncytium formation etc. Membrane blebbing and the translocation of phosphatidi lserine to the cell surface, as well as nuclear concentration and fragmentation, were all observed in PEDV infected cells.

Mechanically, PEDV SM98 and CV777 virus infection induced a significant cleavage on AIFM1 suggested an involvement of mitochondrial-mediated apoptosis pathway, which is consistent with previous observation [19]. Besides this pathway, we found the extrinsic apoptotic pathway was also activated by SM98 and CV777 infection as caspase-8, caspase3 and PARP were all cleaved in response to PEDV virus infection. This study enriched the knowledge of the PEDV infection induced apoptosis through a complicated pattern, apart from the mitochondrial-mediated apoptosis pathway, the extrinsic apoptotic pathway was also activated, no matter whether or not it is original. Consistently, many viruses from coronavirus family induced apoptosis with caspase activation: such as TGEV could cause infected ST cells apoptosis in a caspase-dependent way, accompanied by DNA cleavage and mitochondrial transmembrane potential change [21]; IBV could cause a caspase-dependent apoptosis, as characterized by chromosomal condensation, DNA fragmentation, caspase-3 activation, and poly (ADP-ribose) polymerase degradation [22]; CCoV, is responsible for enteric disease (diarrhea, vomiting, dehydration, loss of appetite and occasional death) in young puppies [23, 24], could cause infected cells apoptosis with activation caspase-3 [23]; SARS-CoV infection could results in the downregulation of Bcl-2, the activation of caspase-3, as well as the upregulation of Bax, suggesting the involvement of the caspase family and the activation of the mitochondrial signaling pathway [25]. MERS-CoV could efficiently infect human primary T lymphocytes and activates the extrinsic and intrinsic apoptosis pathway [26]. However, the exact mechanism especially the critical viral inducer of PEDV has not yet illustrated.

To date, PEDV has evolved into two distinct clades, the classical strains and high-virulent field strains [14]. Antigenic variations of PEDV spike protein (S) between these two groups were thought to contribute to the severity of recent outbreaks [27, 28], as the S protein plays a pivotal role in cell adsorption, membrane fusion, and induction of neutralizing antibodies [29, 30]. Here we demonstrate that S1 protein indeed is a predominant apoptosis inducer (while S2 is not) among all the structural and nonstructural proteins of PEDV, although M and Nsp1, 2, 5, 6, 7, 9 and 13 also induce cell apoptosis but to a less extent. Furthermore, the S1 protein of circulating variant strain PEDV BJ2011 also induce significant apoptosis in host cells, and the ability to induce apoptosis in Vero-E6 is greater than that of the cell adapted strain, such as CV777. This suggested S1 protein is a critical protein mediated PEDV-induced cell apoptosis and contributes partly to its virulence, which is consistent with our laboratory’s recent report [31]. Among all the identified proteins capable inducing apoptosis in host cells, the M protein is the most abundant component of the viral envelope and plays a central role in virus morphogenesis and assembly via its interactions with other viral proteins [32]. The pro-apoptotic role of the SARS-CoV M protein has been revealed by disrupting the interaction of PDK1 with PKB/Akt[33, 34]. This suggests that PEDV M protein may also activate apoptosis through this pathway. 3C-like protease (3CLpro) has been demonstrated could cause cell growth arrest and apoptosis in SARS-CoV 3CLpro-expressing human promonocyte cells [35]. Similarly, the pro-apoptotic function of PEDV Nsp5 i.e. 3C-like protease also has observed in this study. Coronavirus Nsp1 is the first N-terminal cleavage product of the pp1a and pp1a/b polyproteins [36]. Coronavirus Nsp1 regulates host cell and virus gene expression [37] that functions as a potent IFN antagonist. PEDV Nsp1 has been demonstrated could block the nuclear translocation of IRF1 and reduce the number of peroxisomes to suppress IRF1-mediated type III IFNs activities [38]. Here we identified a novel function of PEDV Nsp1 as an inducer of apoptosis.

Coronaviruses can infect a wide range of mammals and birds, but exhibit a marked tropism for epithelial cells of the respiratory and enteric tracts, as well as for macrophages [16, 39], and most of them can induce apoptosis in infected host cells. Although a number of cellular mechanisms and gene products (such as the SARS-CoV M, N protein[34, 40] and 3C-like protease (3CLpro) [35]) capable of inducing apoptosis have been revealed, the S1 protein of these coronaviruses have not yet well-evaluated. Although the amino acid sequence of these S proteins are divergent greatly (Additional file 1: Table S1), here we demonstrated that S1 proteins of all these coronaviruses tested in this study were able to induce apoptosis in transfected Vero-E6 cells. Given the fact that a number of coronaviruses gene products could induce apoptosis in host cells, it is proposed that the spike protein S1 may function as a cofactor that could enhance or stimulate extrinsic apoptotic pathway in these coronavirus induced apoptosis.

## Conclusion

PEDV Spike protein S1 is one of the most critical functional proteins that contributes to cell apoptosis,.Expression of S1 proteins of the coronavirus tested in this study could all induce cell apoptosis suggesting S1 maybe is an effective inducer in Coronavirus-induced apoptosis and targeting S1 protein expression probably is a promising strategy to inhibit coronavirus infection and thus mediated apoptosis on host cells.

## Additional files

Additional file 1: **Table S1.** Primers for genes cloning and amplication. (DOCX 19 kb)

Additional file 2: **Figure. S1.** Fluorescent identification of recombinant plasmids. (PPTX 2490 kb)

Additional file 3: **Figure S2.** Homology and phylogenetic tree analysis of S proteins utilized in the study. (PPTX 151 kb)

## Abbreviations

AIFM1: Apoptosis-inducing factor mitochondria-associated 1
CCoV: Canine coronavirus
FACS: Fluorescence-activated cells sorter
IBV: Infectious bronchitis virus
mAb: Monoclonal antibody
MERS: Middle East respiratory syndrome
Nsp: Non-structural protein
ORF: Open reading frame
PARP: Poly ADP-ribose polymerase
PBS: Phosphate buffered saline
PEDV: Porcine reproductive and respiratory syndrome virus
RT: Room temperature
SARS: Severe acute respiratory syndrome
TGEV: Transmissible gastroenteritis virus
