# Supplementary material for: Porcine epidemic diarrhea virus S1 protein is the critical inducer of apoptosis
Source: Virol J. 2018 Nov 7;15:170. doi: 10.1186/s12985-018-1078-4 (PMC6222994; doi:10.1186/s12985-018-1078-4)
Supplement: Supplementary file 1 — Table S1. Primers for genes cloning and amplication. (DOCX 19 kb) [file 12985_2018_1078_MOESM1_ESM.docx]

**Table S1 Primers for PEDV genes cloning and amplification**

| Primers | sequence（5’→3’） | Construction vectors |
| --- | --- | --- |
| Nsp1-F | ccgctcgagatggctagcaaccatg | pEGFP-Nsp1 |
| Nsp1-R | cgcggatcccgaccaccacgacgac |  |
| Nsp2-F | ccgctcgagatgaacatcgtgccagttgac | pEGFP-Nsp2 |
| Nsp2-R | cgcggatcccgaccacctttcttcttaaaacag |  |
| Nsp3-F | ccgctcgagatgggtgatgttaaattctc | pEGFP-Nsp3 |
| Nsp3-R | cgcggatcccgacccttcttatttgc |  |
| Nsp4-F | ccgctcgagatggcaggtcttcctag | pEGFP-Nsp4 |
| Nsp4-R | cgcggatcccgctgtagagttgaattg |  |
| Nsp5-F | ccgctcgagatggctggcttgcgtaagatg | pEGFP-Nsp5 |
| Nsp5-R | cgcggatcccgctgaagattaacgcca |  |
| Nsp6-F | ccgctcgagatgggtggctatgtgtcac | pEGFP-Nsp6 |
| Nsp6-R | cgcggatcccgctgaacggaagaaatc |  |
| Nsp7-F | ccgctcgagatgtctaaactgactgat | pEGFP-Nsp7 |
| Nsp7-R | cgcggatcccgctgcaacatactattg |  |
| Nsp8-F | ccgctcgagatgagtgttgcatctacttatg | pEGFP-Nsp8 |
| Nsp8-R | cgcggatcccgctggagcttaacaatacg |  |
| Nsp9-F | ccgctcgagatgaataatgagattattcc | pEGFP-Nsp9 |
| Nsp9-R | cgcggatcccgctgcaagcgtacagtgg |  |
| Nsp10-F | ccgctcgagatggctggtaaacaaacag | pEGFP-Nsp10 |
| Nsp10-R | ccggaattcgttgcataatggatc |  |
| Nsp11-F | ccgctcgagatgagcactgatatggcttatttaaaccgagtacggggctc | pEGFP-Nsp11 |
| Nsp11-R | cgcggatcccgttgtaaaactgcagatttctc |  |
| Nsp12-F | ccgctcgagatgcgagtacggggctctagtgc | pEGFP-Nsp12 |
| Nsp12-R | cgcggatcccgttgtaaaactgcagatttctc |  |
| Nsp13-F | ccgctcgagatgtctgcagggctttgtgt | pEGFP-Nsp13 |
| Nsp13-R | cgcggatcccgctgcaaatcagaca |  |
| Nsp14-F | ccgctcgagatggctaatgagggttgtg | pEGFP-Nsp14 |
| Nsp14-R | cgcggatcccgttgcaaattgttactaaatg |  |
| Nsp15-F | ccgctcgagatgggtcttgagaacattgc | pEGFP-Nsp15 |
| Nsp15-R | cgcggatcccgttgaagttgtggataaaatgtc |  |
| Nsp16-F | ccgctcgagatggccagtgaatggaagtgtgg | pEGFP-Nsp16 |
| Nsp16-R | cgcggatcccgtcatttgtttacgttgacc |  |
| S1-F | ccgctcgagatgagggtctttaatttacttctggt | pEGFP-S1 |
| S1-R | cgcggatcccggtaccaccctgccacttgcagg |  |
| S2-F | ccgctcgagatgcaaaaaaggtctgtt | pEGFP-S2 |
| S2-R | ccggaattcgagcttcgtaaggttgaag |  |
| M-F | ccgctcgagatgtctaacggttc | pEGFP-M |
| M-R | ccggaattcggactaaatgaagcac |  |
| N-F | ccgctcgagatggcttctgtcag | pEGFP-N |
| N-R | ccggaattcgttcctgtatcg |  |
| E-F | ccgctcgagatgctacaattagtgaatg | pEGFP-E |
| E-R | ccggaattcgtacgtgaataacag |  |
| ORF3-F | ccgctcgagatgttggcagcgcgttttgc | pEGFP-ORF3 |
| ORF3-R | ccggaattcgttcactaattgtagc |  |
| CV777S1F | ccgctcgagctatgagggtctttaatttacttctggt | pEGFP-CV777S1 |
| CV777S1R | cgggatccctaatggtagaagaaacc |  |
| BJ2011S1F | ccgctcgagctatgaagtctttaacctacttc | pEGFP-BJ2011S1 |
| BJ2011S1R | cgggatccctaatggtagaagaaacc |  |
| IBVS1F | ccggaattcatgttggtaacacctctttta | pEGFP-IBV-S1 |
| IBVS1R | cgcggatccacgtctaaaacgacgtg |  |
| TGEVS1F | ccggaattcatgaaaaaactatttgtggt | pEGFP-TGEVS1 |
| TGEVS1R | cgcggatcctcaatttgtgtaattatatatagag |  |
| CCoVS1F | ccggaattcatgattgtgctaatattgt | pEGFP-CCoVS1 |
| CCoVS1R | cgcggatcctcaattggtataattatatatag |  |
| MERS1F | ccgctcgagctatgatacactcagtgtttc | pEGFP-MERSS1 |
| MERS1R | ccggaattctcattcagatgttt |  |
| SARSS1F | ccgctcgagctatgtttattttcttattatttc | pEGFP-SARSS1 |
| SARSS1R | ccggaattctcattctttatca |  |

**F denotes forward PCR primer; R denotes reverse PCR primer.**
