# Supplementary material for: Porcine epidemic diarrhea virus S1 protein is the critical inducer of apoptosis
Source: Virol J. 2018 Nov 7;15:170. doi: 10.1186/s12985-018-1078-4 (PMC6222994; doi:10.1186/s12985-018-1078-4)
Supplement: Supplementary file 2 — Figure. S1. Fluorescent identification of recombinant plasmids. (PPTX 2490 kb) [file 12985_2018_1078_MOESM2_ESM.pptx]

## Slide 1
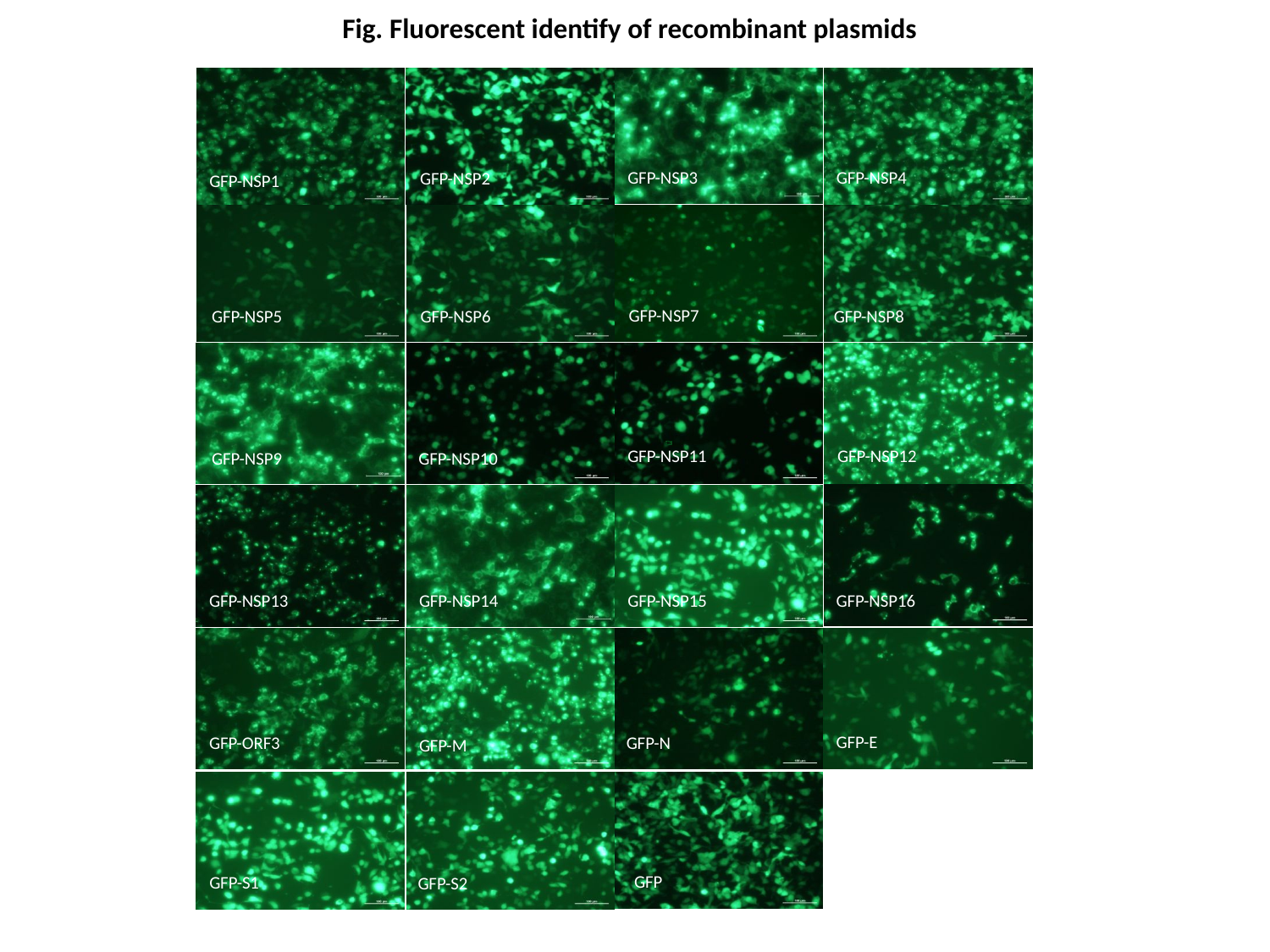

Fig. Fluorescent identify of recombinant plasmids
GFP-NSP4
GFP-NSP3
GFP-NSP2
GFP-NSP1
GFP-NSP7
GFP-NSP6
GFP-NSP5
GFP-NSP8
GFP-NSP11
GFP-NSP12
GFP-NSP10
GFP-NSP9
GFP-NSP13
GFP-NSP14
GFP-NSP16
GFP-NSP15
GFP-E
GFP-ORF3
GFP-N
GFP-M
GFP
GFP-S1
GFP-S2
