# Supplementary figures and images for: Porcine epidemic diarrhea virus S1 protein is the critical inducer of apoptosis
Source: Virol J. 2018 Nov 7;15:170. doi: 10.1186/s12985-018-1078-4 (PMC6222994; doi:10.1186/s12985-018-1078-4)

## Slide 1
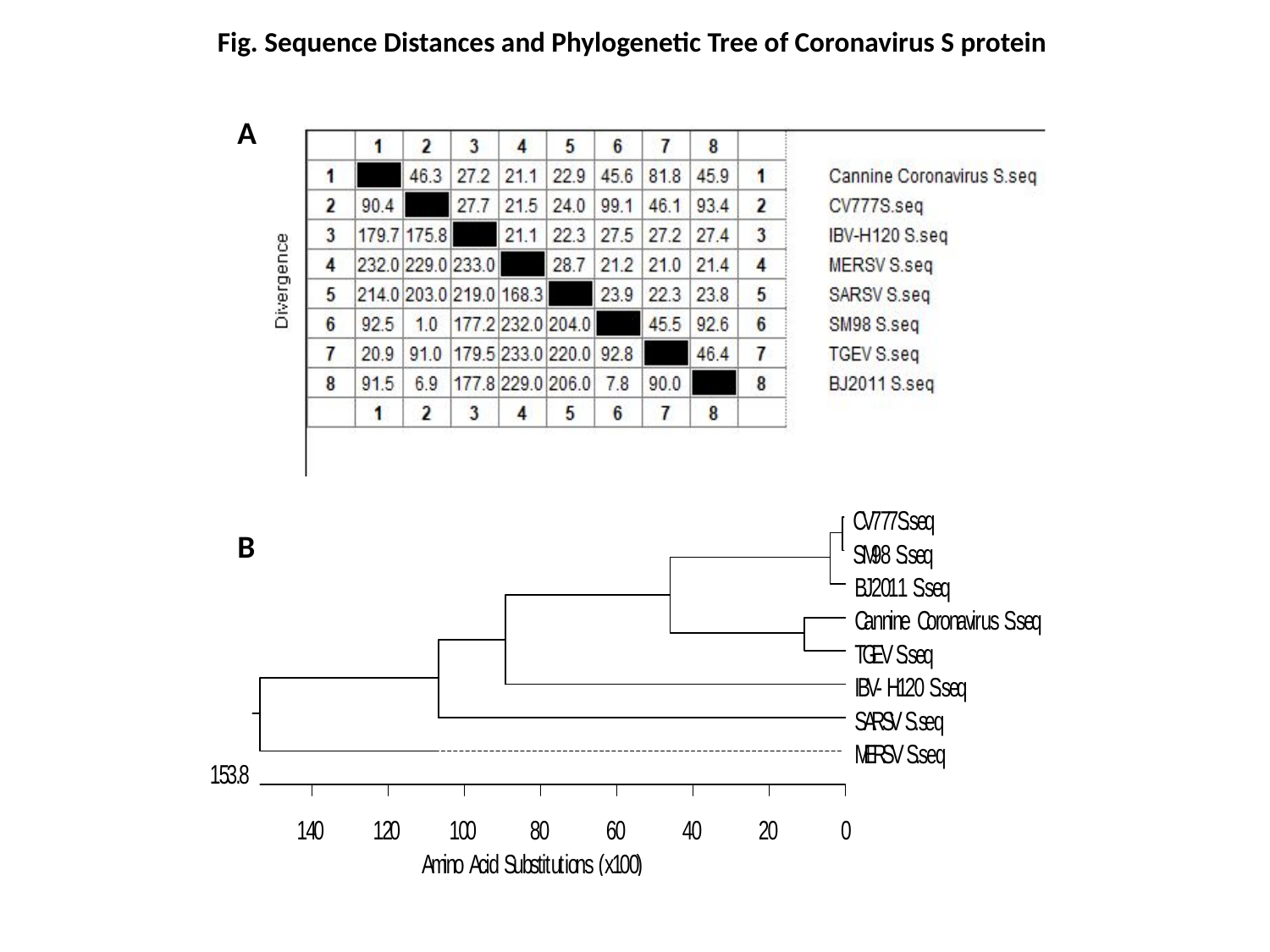

Fig. Sequence Distances and Phylogenetic Tree of Coronavirus S protein
A
B

Supplement: Supplementary file 3 — Figure S2. Homology and phylogenetic tree analysis of S proteins utilized in the study. (PPTX 151 kb) [file 12985_2018_1078_MOESM3_ESM.pptx]
